# Host Response Markers of Inflammation and Endothelial Activation Associated with COVID-19 Severity and Mortality: A GeoSentinel Prospective Observational Cohort

**DOI:** 10.3390/v16101615

**Published:** 2024-10-15

**Authors:** Andrea M. Weckman, Sarah Anne J. Guagliardo, Valerie M. Crowley, Lucia Moro, Chiara Piubelli, Tamara Ursini, Sabrina H. van Ierssel, Federico G. Gobbi, Hannah Emetulu, Aisha Rizwan, Kristina M. Angelo, Carmelo Licitra, Bradley A. Connor, Sapha Barkati, Michelle Ngai, Kathleen Zhong, Ralph Huits, Davidson H. Hamer, Michael Libman, Kevin C. Kain

**Affiliations:** 1 UHN-Toronto General Hospital, University of Toronto, Toronto, ON M5G 1L7, Canada; weckman.andrea@gmail.com (A.M.W.);; 2 Division of Global Migration and Quarantine, Travelers’ Health Branch, Atlanta, GA 30322, USA; 3 Department of Infectious Tropical Diseases and Microbiology, IRCCS Sacro Cuore Don Calabria Hospital, 37024 Negrar di Valpolicella, Italy; 4 Department of Internal Medicine, Antwerp University Hospital (UZA), 2650 Antwerp, Belgium; 5 Department of Clinical and Experimental Sciences, University of Brescia, 25121 Brescia, Italy; 6 International Society of Travel Medicine, Atlanta, GA 30338, USA; 7 Orlando Health Travel Medicine and Infectious Disease, Orlando, FL 34761, USA; 8 Weill Cornell Medical College and the New York Center for Travel and Tropical Medicine, New York, NY 10022, USA; 9 J.D. MacLean Centre for Tropical Diseases, McGill University, Montreal, QC H3A 0G4, Canada; 10 Institute of Tropical Medicine Antwerp, 2000 Antwerp, Belgium; 11 Section of Infectious Diseases, Department of Medicine, Boston University Chobanian & Avedisian School of Medicine, Boston, MA 02118, USA; 12 Center on Emerging Infectious Diseases, Boston University, Boston, MA 02118, USA; 13 Department of Global Health, Boston University School of Public Health, Boston, MA 02118, USA; 14 Division of Infectious Diseases, Department of Medicine, MaRS Centre, TMDT, University of Toronto, 10th Floor 10-351, Toronto, QC M5G 1L7, Canada

**Keywords:** COVID-19, host response, morbidity and mortality, sTREM-1, suPAR

## Abstract

Background: The effect of the COVID-19 pandemic on healthcare systems emphasized the need for rapid and effective triage tools to identify patients at risk of severe or fatal infection. Measuring host response markers of inflammation and endothelial activation at clinical presentation may help to inform appropriate triage and care practices in patients with SARS-CoV-2 infection. Methods: We enrolled patients with COVID-19 across five GeoSentinel clinical sites (in Italy, Belgium, Canada, and the United States) from September 2020 to December 2021, and analyzed the association of plasma markers, including soluble urokinase-type plasminogen activator receptor (suPAR), soluble tumor necrosis factor receptor-1 (sTREM-1), interleukin-6 (IL-6), interleukin-8 (IL-8), complement component C5a (C5a), von Willebrand factor (VWF-a2), and interleukin-1 receptor antagonist (IL-1Ra), with 28-day (D28) mortality and 7-day (D7) severity (discharged, hospitalized on ward, or died/admitted to the ICU). Results: Of 193 patients, 8.9% (16 of 180) died by D28. Higher concentrations of suPAR were associated with increased odds of mortality at D28 and severity at D7 in univariable and multivariable regression models. The biomarkers sTREM-1 and IL-1Ra showed bivariate associations with mortality at D28 and severity at D7. IL-6, VWF, C5a, and IL-8 were not as indicative of progression to severe disease or death. **Conclusions**: Our findings confirm previous studies’ assertions that point-of-care tests for suPAR and sTREM-1 could facilitate the triage of patients with SARS-CoV-2 infection, which may help guide hospital resource allocation.

## 1. Introduction

The COVID-19 pandemic has highlighted how healthcare systems can be rapidly overwhelmed and may be forced to make critical decisions regarding resource allocation. Although most SARS-CoV-2 infections are self-limiting and can be managed at home, some can progress to multi-organ failure and death [1]. Healthcare settings require effective, inexpensive, evidence-based triage tools that can accurately discriminate between patients requiring hospital admission and urgent care versus those that could be safely discharged home. Such tools could lead to decompressed healthcare facilities, decreased nosocomial infections, reduced infections in healthcare workers, and prioritized resource allocation by avoiding inappropriate admissions, as well as improved survival and long-term outcomes by enabling early identification and intervention for those who would otherwise progress to severe disease.

In response to the critical need for SARS-CoV-2 infection triage tools, there was an emergence of an unprecedented number of clinical algorithms for clinical risk stratification [2]. Many algorithms used clinical data alone and could not reliably predict those at risk of severe or fatal infection. However, there is a growing body of evidence that indicates that various life-threatening infections share common pathways of host response that lead to end-organ injury and death [3,4,5,6,7]. Multiple studies have demonstrated that these markers of host response (e.g., inflammation and endothelial activation such as soluble tumor necrosis factor receptor-1 [sTREM-1] and soluble urokinase-type plasminogen activator receptor [suPAR]) may perform well in identifying those at risk of morbidity and mortality in the context of fever and infection due to multiple causes, including SARS-CoV-2 [8,9,10,11]. These markers have often performed better than clinical predictors or scores alone [8,11]. Taken together, these findings suggest that measuring these markers at clinical presentation could facilitate triage, risk stratification, and precision management.

The objective of this project was to implement biomarker surveillance and define their association at admission with progression to severe illness and death in patients with COVID-19 to inform appropriate triage and care practices using patients who presented to GeoSentinel sites. Further, we evaluated the ability of these biomarkers to assess evidence of organ dysfunction (e.g., oxygen requirement), admission to the intensive care unit (ICU), and morbidity at day 7 (D7) and day 28 (D28) post-presentation.

## 2. Methods

### 2.1. Data Source and Ethics

GeoSentinel, a collaboration between the US Centers for Disease Control and Prevention (CDC) and the International Society of Travel Medicine (ISTM), is a global clinical-care-based surveillance and research network that monitors infectious diseases and other adverse health events that may impact international travelers and migrants. GeoSentinel comprises 71 clinical sites in 29 countries on six continents, where clinicians diagnose and treat patients and collect other relevant data. This protocol was reviewed by institutional review boards at all engaged institutions and conducted in compliance with applicable federal law and CDC policy. 

### 2.2. Patient Consent Statement

Written informed consent was obtained for all participants. 

### 2.3. Inclusion Criteria

Adults 18 years of age or older who tested positive for SARS-CoV-2 via polymerase chain reaction (PCR) and were able to provide written informed consent were eligible for inclusion.

### 2.4. Data Collection

#### 2.4.1. Definitions

Acute kidney injury (AKI) was defined as a plasma or serum creatinine > 3 mg/dL or blood urea > 20 mM. Acute lung injury/acute respiratory distress syndrome (ALI/ARDS) was defined as acute onset respiratory failure with a ratio of partial pressure of arterial oxygen to fraction of inspired oxygen (PaO_2_/FiO_2_) < 200, with bilateral infiltrates on a chest radiograph, and no clinical evidence of left atrial hypertension. Quick Sequential Organ Failure Assessment (qSOFA) is a clinical score calculated by assigning a value of one for each of the following signs to develop a score ranging from zero to three: one point each for low blood pressure (systolic blood pressure < 100 mmHg); high respiratory rate (22 breaths per minute); or altered mentation (GCS < 15). 

#### 2.4.2. Study Design

Patients were recruited from five GeoSentinel sites in Europe and North America from September 2020 through December 2021. Data were collected at presentation, on days 3–5, D7, and D28 via RedCap data collection instruments. Venipuncture was performed on all patients on presentation and for hospitalized patients or patients seen in-person for follow-up on days 3–5. Follow-up phone calls were conducted on D7 and D28 if needed. Routine laboratory parameters, including CRP and D-dimer, were analyzed at each academic hospital laboratory. Therefore, some variations in testing methodology are likely and small variabilities across sites are expected. 

#### 2.4.3. Plasma Marker Testing

Blood samples were collected from each participant by venipuncture. EDTA plasma samples were stored at −80 °C and collectively shipped on dry ice to University Health Network in Toronto, ON, Canada, for biomarker analysis. All biomarker assays were conducted by researchers with experience using Luminex, who were blinded to the outcome, in one laboratory at the University Health Network.

Endothelial and inflammatory markers (collectively referred to as “biomarkers”) were selected based on previous studies indicating their use in COVID-19-related morbidity and mortality [11,12,13], or emerging research indicating a role for the marker in the pathogenesis of SARS-CoV-2 [6,14,15]. A Luminex multiplex assay (R&D Systems, Minneapolis, MN, USA; Kit #: LXSAHM-07) was used to measure the plasma concentration of sTREM-1, interleukin-6 (IL-6), interleukin-8 (IL-8), complement component C5a (C5a), von Willebrand factor (VWF-a2), and interleukin-1 receptor antagonist (IL-1Ra). Plasma concentrations of suPAR were quantified using the suPARnostic ELISA kit (ViroGates, Copenhagen, Denmark). A total of 10% of samples were analyzed in duplicate with an average CV of 6%. We were unable to analyze all samples in duplicate due to resource and sample volume constraints.

#### 2.4.4. Statistical Analysis

Data were entered into a REDCap database (version 12.0.8) on a secured CDC server. Statistical analyses of clinical data were conducted using R version 4.0.3 (R Core Team, 2020; Vienna, Austria). 

There were two main dependent variables of interest: D28 mortality, as a final assessment of patient status, and D7 severity score, which captured morbidity earlier during the course of illness. D7 severity was defined as an ordinal variable (discharged < hospitalized on ward < death/ICU).

Patient demographic, clinical, and laboratory characteristics were compared by survival or death at D28 using chi-square/Fisher’s exact tests for categorical data or *t*-tests or Wilcoxon tests for quantitative variables. We also compared biomarker concentration by D28 mortality (Wilcoxon tests) and by D7 severity score (Kruskal–Wallis tests). 

Univariable and multivariable regression models were developed to explore the relationships between mortality (day 28, logistic regression) or morbidity (D7 severity, ordinal regression) and biomarkers, demographic traits, and clinical characteristics that had significant bivariate associations. Only significant univariable factors were included in the multivariable models. Biomarker concentrations were log-transformed prior to modeling. For the ordinal model predicting D7 severity, standard clinical indicators of disease severity were excluded (e.g., oxygen saturation, respiratory rate, lymphocyte count) because these measures would have already been incorporated into the clinical decision to admit a patient to the ICU vs. the ward. 

Model selection was conducted with the StepAIC function in the MASS package in R using forward and backward selection [16]. This method compares the relative improvements in the Akaike Information Criterion (AIC) when adding or dropping each independent variable. The final models were selected on the basis of parsimony and interpretability of the variables. 

For biomarkers that were statistically significant in both multivariable regression models, we defined optimal cut-points using the Youden Index [17], which calculates the difference in sensitivity (true positive rate) and specificity (false positive rate) across all possible cut-points. The Youden Index was calculated based on mortality at D28; values range from 0 (no diagnostic value) to 1 (a perfect test with no false positives or false negatives).

## 3. Results

### 3.1. Characteristics of the Cohort

**Figure 1:**
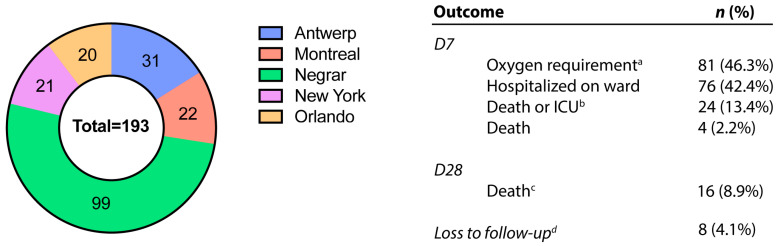
Number of included patients with COVID-19 infection by study site and patient outcome: GeoSentinel (2020–2021). Abbreviations: intensive care unit (ICU). *n* (%) as a proportion of patients with existing data. ^a^ Any oxygen requirement (1 to 6 liters, bilevel positive airway pressure [BIPAP] or continuous positive airway pressure [CPAP], intubated); ^b^ death or hospitalized in ICU at day 7; ^c^ death before 28 days, including *n* = 4 deaths that occurred before day 7; ^d^ *n*= 8 participants missing outcome data at both D7 and D28. An additional six (total *n* = 14) participants were missing outcome data only at D7, and five (total *n* = 13) participants were missing outcome data only at D28 endpoint.

One hundred and ninety-three patients were enrolled (Negrar, Italy [*n* = 99, 51.3%], Antwerp, Belgium [*n* = 31, 16.1%], Montreal, Canada [*n* = 22, 11.4%], New York, USA [*n* = 21, 10.9%], and Orlando, USA [*n* = 20, 10.4%]). Of the total cohort, 81 (46.3%) patients required oxygen by D7 and four (2.2%) patients died by D7. By D28, we recorded 16 deaths (8.9%). Eight (4.1%) participants were completely lost to follow-up, with no outcome data at D7 or D28 (Figure 1). 

The median age of patients was 63 years (range: 22–102), and 39.9% were female (Table 1). Patients had a variety of comorbidities, most frequently chronic cardiac disease (27.1%) and obesity (26.6%). On clinical presentation, most patients were normotensive, afebrile, and had a regular heart rate. However, median respiratory rates and oxygen saturations were abnormal; forty-three patients (22.8%) presented with ALI/ARDS. Inflammatory markers including C-reactive protein and D-dimer were elevated. Differences in demographics, clinical findings, and laboratory results across GeoSentinel sites are shown in Supplementary Table S1.

### 3.2. Mortality at D28

**Figure 2:**
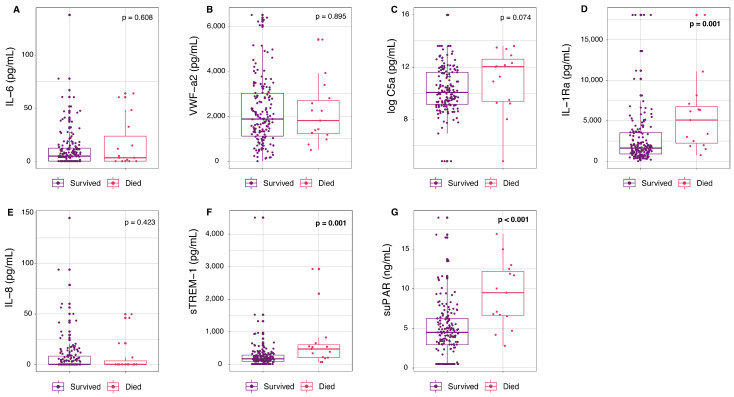
Circulating concentrations of inflammatory and endothelial markers at presentation in patients with COVID-19 by mortality outcome at D28: GeoSentinel (2020–2021). Circulating concentrations of inflammatory and endothelial markers at inclusion including (**A**) IL-6, (**B**) VWF-a2, (**C**) C5a, (**D**) IL-1Ra, (**E**) Il-8, (**F**) sTREM-1, and (**G**) suPAR stratified by mortality outcome at D28. Survived includes those who were discharged home (*n* = 141), discharged to hospice/long-term care (*n* = 4), or were in hospital (*n* = 19) at D28. Deaths include those who died before 7 days (*n* = 4) and deaths between 7 and 28 days (*n* = 12). Concentrations of C5a (**C**) have been logged for visualization. Significant *p*-values (*p* < 0.05 by Wilcoxon rank sum test) are in bold. Abbreviations: complement component C5a (C5a), interleukin-1 receptor antagonist (IL-1Ra), interleukin-6 (IL-6), interleukin-8 (IL-8), soluble tumor necrosis factor receptor-1 (sTREM-1), soluble urokinase-type plasminogen activator receptor (suPAR), von Willebrand factor (VWF-a2).

Patients who died by D28 had an older median age (76 years vs. 62 years, *p* < 0.001), higher rates of chronic cardiac disease (62.5% vs. 24.5%, *p* = 0.003), and higher rates of ALI or ARDS (56.3% vs. 20.6%, *p* = 0.006) at enrollment in the trial, compared to those that survived (Table 1). Laboratory results showed that those who died by D28 had lower lymphocyte counts (0.7 × 10^9^/L vs. 0.9 × 10^9^/L, *p* < 0.001) and higher C-reactive protein levels (122.6 mg/L vs. 62.3 mg/L, *p* = 0.049) compared to those who survived (Table 1). Mortality before D28 was also associated with an increased concentration of suPAR (*p* < 0.001), IL-1Ra (*p* = 0.001), and sTREM-1 (*p* = 0.001) at presentation by bivariate analysis (Figure 2).

The log-transformed concentrations for suPAR (odds ratio [OR] = 8.4, 95% CI: 2.7–29.4, *p* < 0.005), sTREM (OR = 2.6, 95% CI: 1.5–5.0, *p* < 0.005), and IL-1Ra (OR = 2.4 [1.4–4.4, *p* < 0.005]) were significant univariable regressors of D28 mortality, along with lymphocyte count, age > 65, ALI or ARDS, chronic cardiac disease rate, and c-reactive protein (Supplementary Table S2). The final multivariable logistic regression model showed that higher values of log-transformed suPAR concentration resulted in increased odds of D28 mortality (adjusted odds ratio [aOR] = 6.0, 95% CI: 1.9–22.9, *p* < 0.01) after adjusting for chronic cardiac disease (aOR = 3.9, 95% CI: 1.2–14.5, *p* < 0.05) and age > 65 (aOR = 9.9, 95% CI: 1.7–187.9, *p* < 0.05) (Table 2).

### 3.3. Severity (Morbidity) at D7 

D7 severity was defined as an ordinal variable (discharged < hospitalized on ward < death/ICU). Patients with more severe outcomes at D7 had an older median age (54 years among those discharged vs. 73 years and 69.5 years among those hospitalized on ward and ICU, respectively, *p* < 0.0001), and higher rates of chronic kidney disease (1.3% [discharged] vs. 9.2% [ward] vs. 16.7% [ICU], *p* < 0.05) (Supplementary Table S3). 

**Figure 3:**
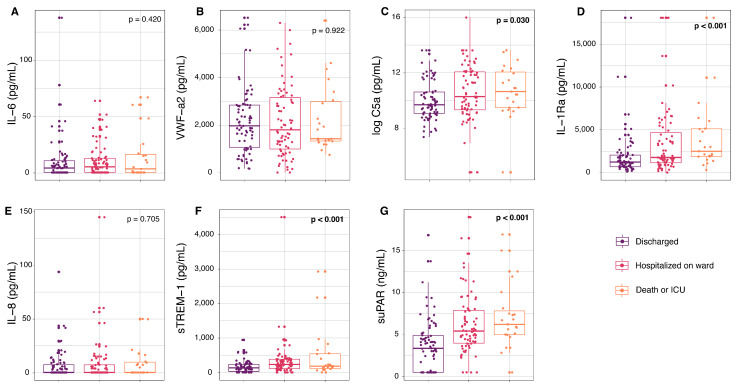
Circulating concentrations of inflammatory and endothelial markers at presentation in patients with COVID-19 by severity outcome at D7: GeoSentinel (2020–2021). Circulating concentrations of inflammatory and endothelial markers at inclusion including (**A**) IL-6, (**B**) VWF-a2, (**C**) C5a, (**D**) IL-1Ra, (**E**) Il-8, (**F**) sTREM-1, and (**G**) suPAR stratified by severity outcome at D7. Discharged at D7 (*n* = 79), hospitalized on ward at D7 (*n* = 76), and death or ICU (*n* = 24) includes those who were admitted to the ICU (*n* = 20) and those who died by D7 (*n* = 4). Concentrations of C5a (**C**) have been logged for visualization. Significant *p*-values (*p* < 0.05 by Kruskal–Wallis) are in bold. Abbreviations: complement component C5a (C5a), interleukin-1 receptor antagonist (IL-1Ra), interleukin-6 (IL-6), interleukin-8 (IL-8), soluble tumor necrosis factor receptor-1 (sTREM-1), soluble urokinase-type plasminogen activator receptor (suPAR), von Willebrand factor (VWF-a2).

At D7, there were significant elevations in circulating concentration of C5a (*p* = 0.030), IL-1Ra (*p* < 0.001), sTREM-1 (*p* < 0.001), and suPAR (*p* < 0.001) across levels of increasing disease severity (Figure 3). Significant univariable factors associated with (ordinal) D7 severity included suPAR (OR = 2.7, 95% CI: 1.9–4.0, *p* < 0.0001), sTREM-1 (OR = 1.6, 95% CI: 1.3–2.0, *p* < 0.0001), and IL-1Ra (OR = 1.6, 95% CI: 1.2–2.2, *p* < 0.0005) (Supplementary Table S4). Age > 65, chronic kidney disease, and BMI were also significant in the univariable models. The best multivariable logistic regression model showed that higher concentrations of (log-transformed) suPAR resulted in increased odds of D7 severity (aOR = 2.2, 95% CI: 1.5–3.3, *p* < 0.0001) after adjusting for age > 65 (aOR = 3.2, 95% CI: 1.6–6.3, *p* <0.005), and the occurrence of chronic kidney disease (aOR = 4.0, 95% CI: 1.0–16.1, *p* < 0.05) (Table 2).

### 3.4. Proposed Cut-Off 

suPAR was the only statistically significant biomarker identified in both multivariable regression models predicting D28 mortality and D7 severity. The suPAR cut-off of 6.525 ng/mL yielded the optimal Youden Index value for D28 mortality (0.57, with a sensitivity of 0.80 and a specificity of 0.77).

## 4. Discussion

This analysis demonstrates that death and severity of infection with SARS-CoV-2 are associated with elevated mediators of inflammatory and endothelial activation including suPAR, sTREM-1, IL1Ra, and C5a. suPAR was the only biomarker that was a statistically significant independent variable in both multivariable models predicting D28 mortality and D7 severity. 

Triage tools that could, at initial clinical presentation, reliably identify patients with impending severe infection requiring referral/admission or ICU care, while safely identifying those with uncomplicated infection, could aid in the management of COVID-19 and other severe infections. An accumulating body of evidence supports markers of inflammation and endothelial activation for use as rapid triage tools to complement and enhance clinically based triage. 

suPAR has resurfaced as an additional generalizable marker of infection severity and morbidity/mortality, with prognostic capacity in infections including HIV-1, bacterial pneumonia, sepsis, malaria, and COVID-19 [11,13,18,19,20,21,22]. In our cohort, suPAR had the strongest association with both D28 mortality and D7 severity. The median suPAR concentration observed in our cohort (9.5 ng/mL in those who died by 28 days vs. 4.5 ng/mL in survivors) aligns quite closely with a recent metanalysis of suPAR data in patients with COVID-19 [13]. Our proposed cut-off of 6.52 ng/mL for D28 mortality aligns well with trials suggesting a cut-off of ≥ 6 ng/mL suPAR concentration for risk stratification and targeted intervention in patients with SARS-CoV-2 infection [18,23,24]. A point-of-care (POC) suPAR test is commercially available, and collectively, these data support its use as a tool to inform risk stratification of patients infected with SARS-CoV-2. 

Neutrophils, monocytes, and macrophages express the cell-surface receptor TREM-1 and its activation triggers amplification of the immune response [25]. The soluble form of TREM-1 (sTREM-1) has emerged as a leading marker for its ability to discriminate between those at risk of morbidity and mortality, those with uncomplicated infection across a range of infections (e.g., pneumonia, malaria, COVID-19, sepsis, dengue), and populations (e.g., adults, children, low- and middle-income countries, high-income countries) [8,10,26]. The wide generalizability of sTREM-1 across infections and populations including immunosuppressed patients makes it a promising tool for rapid identification of those at risk of severe disease. Although sTREM-1 was not selected in the final multivariable models, bivariate and univariable associations with both D28 mortality and D7 severity, along with existing evidence in the literature, indicate sTREM-1 cannot be ruled out as a useful triage tool at hospital presentation in patients infected with SARS-CoV-2. 

The cytokine storm and immunothrombogenesis have been implicated in the pathobiology of COVID-19, including excessive activation of the IL-1, IL-6, IL-8, complement, and coagulation pathways (reviewed in [6,27,28]). Here, we investigated the prognostic capabilities for components of these pathways (i.e., IL-1Ra, IL-6, IL-8, VWFa2), with varying results. Of these markers, IL-1Ra (an anti-inflammatory modulator of the IL-1 pathway) was the only marker that displayed a moderate ability to identify those at risk of 28-day mortality in our cohort (i.e., in bivariate and univariable regression models), externally validating a small amount of existing data [15,29]. While C5a did not have a high level of prognostic accuracy for mortality at day 28, in bivariate analyses, it was significantly elevated in those with increasing disease severity at day 7. This may indicate C5a, as an anaphylatoxin, is more suited for indicating immediate rather than long-term disease severity, supported by existing experimental data [14,28]. Despite evidence indicating elevated IL-6 and VWF in COVID-19, and some data supporting the use of IL-6 as a prognostic indicator, especially for oxygen requirement [11,12,30], neither IL-6 nor VWF were indicative of progression to severe disease in our cohort. Like C5a, there is also evidence that IL-6 may be more useful as an indicator of immediate disease severity and the cytokine storm [15] rather than as an early predictor of mortality. 

Collectively, these data support a pattern that suPAR appears to be the most consistently effective marker for early identification of those at risk of progression to severe disease and death in patients with COVID-19. Other markers including sTREM-1 and IL-6 may be more dependent on patient population or severity of disease at presentation. In a cohort with a relatively high morbidity and mortality rate (46% D7 oxygen requirement and 9% D28 mortality), our analysis provides a validation for the use of suPAR as a putative POC test for identification of patients with SARS-CoV-2 infection at risk of severe disease and death. Our study was strengthened by its multi-site, international design, indicating further generalizability of these markers for triage.

This analysis has a couple of limitations. First, GeoSentinel sites are clinical sites that focus on travel medicine, so patients may not be representative of non-traveling patients; however, given minimal international travel during the project’s timeframe, sites were directly involved in the care of non-traveler populations and most of our participants were non-travelers. Second, generalizability and statistical power are also limited by the relatively small sample size and the interval nature of our outcomes. Future work in this area could include a survival analysis to assess the relationship between exact length of survival and COVID-19 biomarkers. In addition, routine laboratory parameters, including CRP and D-dimer, were analyzed at each individual academic site laboratory rather than a central location, which may have introduced some variability in these measurements across sites. 

In summary, we present additional data validating the use of suPAR as a tool to guide risk stratification of patients presenting with SARS-CoV-2 infection. POC tests for suPAR are commercially available, enabling prospective trials to guide risk stratification for COVID-19 as well as other potentially severe infections. 

## Figures and Tables

**Table 1:** Demographics, clinical findings, and laboratory results of patients with COVID-19 on presentation stratified by mortality at study endpoint (D28): GeoSentinel (2020–2021).

	Entire Cohort ^a^	Survived ^b^	Died ^c^	*p*-Value ^d^
*n*	193	164 (91.1%)	16 (8.9%)	--
Demographics				
Median age in years, (range)	63 (22, 102)	62 (22, 102)	76 (60, 94)	<0.001 *
Female sex at birth (%)	77 (39.9)	63 (38.4)	7 (43.8)	0.881
Median BMI (kg/m^2^), (IQR)	27.9 (25.1, 31.3)	28.1 (25.9, 31.2)	24.1 (22.1, 31.9)	0.120 *
Comorbidities, *n* (%)				
Diabetes	45 (23.8)	37 (23.0)	6 (37.5)	0.324
Asthma	14 (7.3)	14 (8.5)	0 (0)	0.697 ^†^
Malnutrition	4 (2.1)	4 (2.5)	0 (0)	1.00 ^†^
Obesity	50 (26.6)	43 (26.9)	5 (31.3)	0.936
Chronic cardiac disease	52 (27.1)	40 (24.5)	10 (62.5)	0.003
Chronic kidney disease	14 (7.3)	10 (6.1)	3 (18.8)	0.165 ^†^
Chronic pulmonary disease	20 (10.5)	14 (8.7)	3 (18.8)	0.331 ^†^
Chronic liver disease	6 (3.1)	4 (2.5)	1 (6.3)	0.533 ^†^
Malignant neoplasm	23 (12.0)	19 (11.7)	4 (25.0)	0.271 ^†^
Clinical findings				
Blood pressure (mmHg)				
Systolic, median (IQR)	130 (117, 140)	130 (118, 140)	132 (119, 136)	0.872 *
Diastolic, mean (SD)	77 (12)	76 (11)	73 (16)	0.452 *
Temperature (°C), median (IQR)	37.0 (36.3, 37.7)	37.0 (36.4, 37.7)	37.1 (36.2, 38.3)	0.656 *
Heart rate (beats per min), median (IQR)	85 (74, 100)	85 (74.8, 100)	90 (79.3, 105)	0.358 *
Respiratory rate (breaths per min), median (IQR)	20 (17, 23)	20 (16, 22)	21 (18, 30)	0.061 *
Oxygen saturation (SpO_2_), median (range)	94 (92, 96)	94 (92, 96)	92 (85, 96)	0.125 *
ALI or ARDS, *n* (%)	43 (22.8)	33 (20.6)	9 (56.3)	0.006 ^†^
Acute kidney injury, *n* (%)	13 (7.0)	10 (6.1)	3 (18.8)	0.147 ^†^
Glasgow Coma Score < 15, *n* (%)	9 (4.7)	6 (3.7)	1 (6.3)	0.656 ^†^
qSOFA Scores, *n* (%)				0.091 ^†^
0	130 (67.7)	114 (69.9)	7 (43.8)	
1	58 (30.2)	45 (27.6)	9 (56.2)	
2	4 (2.0)	4 (2.5)	0 (0.0)	
Laboratory results				
Platelet count (10^9^/L), median (IQR)	210 (164, 268)	213 (164, 265)	196 (151, 292)	0.608 *
White blood cell count (10^9^/L), median (IQR)	6.5 (4.4, 9.6)	6.4 (4.4, 9.6)	7.1 (4.3, 7.9)	0.988 *
Lymphocyte count (10^9^/L), median (IQR)	0.9 (0.7, 1.4)	0.9 (0.7, 1.4)	0.7 (0.4, 0.8)	<0.001 *
Neutrophil count (10^9^/L), median (IQR)	4.9 (2.8, 7.9)	4.8 (2.9, 8.0)	5.9 (3.0, 6.7)	0.587 *
Neutrophil-to-lymphocyte ratio, median (IQR)	4.7 (2.7, 9.0)	4.6 (2.7, 8.8)	10.1 (5.0, 15.5)	0.007 *
C-reactive protein (mg/L), median (IQR)	63.5 (21.5, 121.0)	62.3 (21.7, 117.2)	122.6 (70.0, 146.0)	0.049 *
D-dimer (mg/L), median (IQR)	0.8 (0.5, 1.2)	0.8 (0.5, 1.2)	1.1 (0.6, 2.1)	0.159 *

Abbreviations: acute lung injury (ALI), acute respiratory distress syndrome (ARDS), body mass index (BMI), interquartile range (IQR), Quick Sequential Organ Failure Assessment (qSOFA). Data are presented as *n* (% of available data) for categorical variables, mean (SD) for normal continuous variables, and median (IQR) for non-normal continuous variables. Age is presented as median (range). ^a^ *n* = 8 with no outcome data at D7 or D28, *n* = 13 lost to follow-up at D28; ^b^ includes those who were discharged (*n* = 145) and those who remain hospitalized but alive (*n* = 19) by D28; ^c^ includes those who died at D7 (*n* = 4) and those who died by D28 (*n* = 12). ^d^ *p*-values are calculated using chi-square tests unless otherwise noted. * *t*-test (normal distribution) or Wilcoxon test (non-parametric distribution). ^†^ Fisher’s exact test *p*-value.

**Table 2:** Multivariable logistic regression model for D28 mortality and D7 severity using patient demographic, clinical, laboratory, and biomarker data at presentation from patients with COVID-19 infection: GeoSentinel (2021–2022).

D28 Mortality				
Characteristic	aOR	(95% CI)	*p*	AIC
log(suPAR [ng/mL])	6.0	(1.9–22.9)	<0.01	78.6
Chronic cardiac disease	3.9	(1.2–14.5)	<0.05	
Age > 65	9.9	(1.7–187.9)	<0.05	
D7 Severity				
Characteristic	aOR	(95% CI)	*p*	AIC
log(suPAR [ng/mL])	2.2	(1.5–3.3)	<0.0001	284.5
Age > 65	3.2	(1.6–6.3)	<0.005	
Chronic kidney disease	4.0	(1.0–16.1)	<0.05	

Abbreviations: adjusted odds ratio (aOR), Akaike Information Criterion (AIC), confidence interval (CI), soluble urokinase-type plasminogen activator receptor (suPAR).

## Data Availability

The original contributions presented in the study are included in the article/supplementary material, further inquiries can be directed to the corresponding author.

## Supplementary Materials

The following supporting information can be downloaded at https://www.mdpi.com/article/10.3390/v16101615/s1, Supplementary Table S1: Patient demographics, clinical findings, and laboratory results at presentation of patients with COVID-19, stratified by GeoSentinel site (2020–2021); Supplementary Table S2: Univariable logistic regression models for D28 mortality using patient demographic, clinical, laboratory, and biomarker data at presentation from patients with COVID-19, GeoSentinel (2021–2022); Supplementary Table S3: Demographics, clinical findings, and laboratory results at presentation of patients with COVID-19, stratified by severity at D7, GeoSentinel (2020–2021); Supplementary Table S4. Univariable ordinal regression models for D7 severity using patient demographic, clinical, and biomarker data at presentation from patients with COVID-19, GeoSentinel (2021–2022).
