# Supplementary material for: Host Response Markers of Inflammation and Endothelial Activation Associated with COVID-19 Severity and Mortality: A GeoSentinel Prospective Observational Cohort
Source: Viruses. 2024 Oct 15;16(10):1615. doi: 10.3390/v16101615 (PMC11512287; doi:10.3390/v16101615)
Supplement: Supplementary file 1 [file viruses-16-01615-s001.zip › viruses-3203923-supplementary.pdf]

Supplementary Table S1. Patient demographics, clinical findings, and laboratory results at presentation of patients with COVID-19, stratified by GeoSentinel site (2020–2021)

	**Antwerp** n = 31	**Montreal** n = 22	**Negrar** n = 99	**New York** n = 21	**Orlando** n = 20	**p-value**
**Demographic**						
Median age in years (range)	55 (22, 84)	56 (23, 102)	71 (32, 93)	49 (24, 75)	51 (22, 84)	**<0.001***
Female sex at birth, n (%)	14 (45.2)	7 (31.8)	30 (30.3)	12 (57.1)	14 (70)	**0.005**
BMI (kg/m^2), median (IQR)	27.3 (25.0, 31.3)	25.6 (21.9, 27.6)	27.8 (25.2, 29.9)	30.3 (28.4, 35.5)	33.3 (26.6, 38.2)	**<0.001***
**Comorbidities, n (%)**						
Diabetes	5 (16.1)	4 (18.2)	24 (24.5)	4 (19.0)	8 (47.1)	**0.002**
Asthma	2 (6.4)	1 (4.5)	2 (2.0)	7 (33.3)	2 (10.0)	**<0.001**
Malnutrition	0 (0)	4 (19.0)	0 (0)	0 (0)	0 (0)	**<0.001**
Obesity	9 (30.0)	5 (23.8)	21 (21.6)	8 (38.1)	7 (36.8)	0.417
Chronic kidney disease	0 (0)	4 (18.2)	6 (6.1)	1 (4.8)	3 (15.0)	0.076
Chronic cardiac disease	3 (9.7)	9 (40.9)	31 (31.6)	2 (9.5)	7 (35.0)	**0.019**
Chronic pulmonary disease	3 (9.7)	7 (31.8)	6 (6.2)	2 (9.5)	2 (10.5)	**0.014**
Chronic liver disease	0 (0)	2 (9.1)	4 (4.1)	0 (0)	0 (0)	0.267
Malignant neoplasm	2 (6.4)	1 (4.5)	17 (17.3)	1 (4.8)	2 (10.0)	0.211
**Clinical**						
Blood pressure (mmHg)<br>Systolic, median (IQR)<br>Diastolic, mean (SD)	 122 (119, 133)<br>73 (9.3)	 120.5 (110, 135)<br>72.6 (12)	 135 (120, 143)<br>76.2 (12)	 126 (117, 134)<br>80.0 (13)	 128 (118, 139)<br>74.7 (10)	 0.225*<br>0.182*
Temperature (°C), median (IQR)	37.1 (36.8, 37.5)	36.5 (36.3, 36.8)	36.9 (36.1, 36.8)	37.6 (37.0, 38.3)	37.2 (36.7, 38.6)	**0.001***
Heart rate (beats per min), median (IQR)	87 (82, 99)	76 (65, 80)	84 (74, 98)	97 (84, 106)	100 (84, 111)	**<0.001***
Respiratory rate (breaths per min), median (IQR)	16 (16, 20)	19 (18, 20)	20 (18, 24)	19 (17, 22)	22 (19, 24)	**0.002***
Oxygen saturation (SpO_2), median (IQR)	95 (94, 97)	96 (94, 97)	93 (91, 96)	94 (92, 96)	91.5 (**76**, 94)	**<0.001***
Acute kidney injury, n (%)	0 (0)	2 (9.1)	8 (8.2)	0 (0)	3 (15.0)	0.207
ALI or ARDS, n (%)	0 (0)	0 (0)	38 (38.4)	0 (0)	5 (25.0)	**<0.001**
Glasgow Coma Score < 15, n (%)	0 (0)	5 (23.8)	2 (2.0)	0 (0)	2 (10.0)	**<0.001**
**Outcomes**						
D7 oxygen requirement[b], n (%)	9 (33.3)	3 (17.6)	54 (55.7)	7 (35.0)	7 (53.8)	**0.020**
D28 mortality[c], n (%)	3 (10.3)	1 (6.7)	12 (12.1)	0 (0)	0 (0)	0.282

Abbreviations: acute lung injury (ALI), acute respiratory distress syndrome (ARDS), body mass index (BMI), interquartile range (IQR), standard deviation (SD). Data presented as n (% of available data for respective variable [i.e., excluding missing data]) for categorical variables, mean (SD) for normal continuous variables, and median (IQR) for non-normal continuous variables. Corresponding p-values represent Pearson's chi-square test, anova, or Kruskal-wallis test, respectively. [b]Oxygen requirement at D7 defined as need for any oxygen including 1 to 6 liters, BPAP/CPAP, or intubation. [c]D28 mortality includes n=4 people who died before D7, and n=12 people who died before D28. Significant p-values are bolded. P-values are chi-square tests unless otherwise noted. *T-test (normal distribution) or Wilcoxon test (non-parametric distribution).

Supplementary Table S2. Univariable logistic regression models for D28 mortality using patient demographic, clinical, laboratory, and biomarker data at presentation from patients with COVID-19, GeoSentinel (2021–2022)

**Characteristic**	**OR**	**(95% CI)**	**P**	**AIC**
log(suPAR [ng/mL])	8.4	(2.7-29.4)	**<0.005**	88.0
log(sTREM-1 [pg/mL])	2.6	(1.5-5.0)	**<0.005**	93.7
Lymphocyte count (10^9/L)	0.05	(0.006-0.3)	**<0.005**	95.6
log(IL-1Ra [pg/mL])	2.4	(1.4-4.4)	**<0.005**	96.5
Age > 65	8.5	(2.3-55.3)	**<0.01**	100.4
ALI or ARDS	4.9	(1.7-14.8)	**<0.005**	104.6
Chronic cardiac disease	5.1	(1.8-15.9)	**<0.005**	102.6
C-reactive protein (mg/L)	1.005	(0.9-1.009)	0.11	106.3

Abbreviations: Akaike Information Criterion (AIC), acute lung injury (ALI), acute respiratory distress syndrome (ARDS), confidence interval (CI), interleukin-1 receptor antagonist (IL-1Ra), odds ratio (OR), soluble urokinase-type plasminogen activator receptor (suPAR). Univariable models are presented in order of significance (yes/no) and lowest AIC.

Supplementary Table S3. Demographics, clinical findings, and laboratory results at presentation of patients with COVID-19, stratified by severity at D7, GeoSentinel (2020–2021)

	Entire cohort at D7[a]	Discharged	Hospitalized on ward	ICU/ Died[b]	p-value
n	179	79 (44.1%)	76 (42.5%)	24 (13.4%)	--
**Demographics**					
Median age in years, (range)	65 (22, 102)	54 (22, 86)	73 (26, 102)	69.5 (23, 94)	**<0.0001***
Female sex at birth (%)	69 (38.5)	33 (41.8)	26 (34.2)	10 (41.7)	0.789
Median BMI (kg/m^2), (IQR)	27.9 (25.3, 31.02)	29.04 (26.1, 33.5)	27.02 (24.4, 29.3)	28.6 (24.4, 33.5)	**<0.005***
**Comorbidities, n (%)**					
Diabetes	40 (22.7)	12 (15.8)	22 (28.9)	6 (25.0)	0.281
Asthma	14 (7.9)	8 (10.3)	5 (6.6)	1 (4.2)	0.833†
Malnutrition	4 (2.3)	0 (0)	3 (4.0)	1 (4.2)	0.221†
Obesity	47 (26.9)	22 (28.9)	14 (18.7)	11 (45.8)	0.0679
Chronic cardiac disease	50 (28.1)	15 (19.2)	24 (31.6)	11 (45.8)	0.0649
Chronic kidney disease	12 (6.7)	1 (1.3)	7 (9.2)	4 (16.7)	**<0.05**†
Chronic pulmonary disease	19 (10.8)	3 (3.9)	12 (16.0)	4 (16.7)	0.0526†
Chronic liver disease	6 (3.4)	1 (1.3)	4 (5.3)	1 (4.2)	0.479†
Malignant neoplasm	23 (12.9)	7 (9.0)	14 (18.4)	2 (8.3)	0.35†

Abbreviations: body mass index (BMI), intensive care unit (ICU), interquartile range (IQR). Data presented as n (% of available data) for categorical variables, mean (SD) for normal continuous variables, and median (IQR) for non-normal continuous variables. Age is presented as median (range). [a] n=14 with no outcome data at D7; [b] Includes those who were admitted to the ICU (n = 20) and those who died by D7 (n=4). Significant p-values are bolded. P-values are chi-square tests unless otherwise noted. *Kruskal-Wallis p-value. †Fisher's exact test p-value.

Supplementary Table S4. Univariable ordinal regression models for D7 severity using patient demographic, clinical, and biomarker data at presentation from patients with COVID-19, GeoSentinel (2021–2022)

Characteristic	OR	(95% CI)	P	AIC
log(suPAR ([ng/mL])	2.7	(1.9-4.0)	**<0.0001**	297.5
Age > 65	4.8	(2.6-9.3)	**<0.0001**	304.8
log(sTREM-1 [pg/mL])	1.6	(1.3-2.0)	**<0.0001**	315.0
log(IL-1Ra [pg/mL])	1.6	(1.2-2.2)	**<0.0005**	317.7
Chronic kidney disease	8.0	(2.2-29.7)	**<0.005**	320.1
BMI (kg/m^2)	0.9	(0.86-0.99)	**<0.05**	325.5
log(C5a [pg/mL])	1.2	(0.9-1.4)	0.07	326.9

Abbreviations: Akaike Information Criterion (AIC), acute lung injury (ALI), acute respiratory distress syndrome (ARDS), complement component C5a (C5a), confidence interval (CI), interleukin-1 receptor antagonist (IL-1Ra), odds ratio (OR), soluble urokinase-type plasminogen activator receptor (suPAR). Univariable models are presented in order of significance (yes/no) and lowest AIC.
